# Radio-Morphometric Analysis of Sella Turcica in Relation to Age and Gender in Sri Ganganagar Population: A Prospective Cephalometric Study

**DOI:** 10.7759/cureus.32048

**Published:** 2022-11-30

**Authors:** Meenakshi P Surana, Basavaraj T Bhagawati, Nishant Kumar, Samreen Jaral, Ankit Kumar, Sharanamma B

**Affiliations:** 1 Dentistry, Darshan Dental Clinic, Thane, IND; 2 Oral Medicine & Radiology, Surendera Dental College & Research Institute, Sri Ganganagar, IND; 3 Periodontics, Hazaribag College of Dental Sciences and Hospital, Hazaribag, IND

**Keywords:** gender, age, morphometric, forensic dentistry, lateral cephalogram, sella turcica

## Abstract

Background

Sella turcica (ST) is a crucial structure that is morphologically situated in the median position and is well-utilised in cephalometrics. This saddle-shaped sella constitutes a significant radiographic landmark for various related analyses. Therefore, studying its varying dimensions in different populations is of utmost importance. This paper evaluates and compares the linear dimensions and morphological variations of ST in different facial skeletal classes in relation to age and gender using lateral cephalograms in the Sri Ganganagar population.

Methodology

The study population was selected through simple random sampling from the accessible population of the Sri Ganganagar district. A total of 180 participants of both genders were selected from patients who visited the outpatient clinic of the Department of Oral Medicine and Radiology of Surendra Dental College and Research Institute in Sri Ganganagar. These samples were equally divided into three age groups. Apart from typical morphology, five possible variations of ST were determined. The area of ST on the lateral cephalogram was also evaluated. The extent of ST was calculated from the tubercle portion to the top of the dorsal side. The relative deepness of the sella was assessed by making a tangent across the innermost point of the sella. SPSS software was utilised for statistical analysis and related inferences.

Results

In the study sample (n = 180), 50% were men and 50% were women. The sample was divided into three equal groups based on age: Group I (n = 60; 33.33%) consisted of participants aged 15-20; Group II (n = 60; 33.33%) consisted of participants aged 21-25; and Group III (n = 60; 33.33%) consisted of participants aged 26-30.

Conclusions

The anteroposterior diameter of the sella structure is strongly related to age progression with no significant gender correlation. The most common shape of ST other than the normal one was oblique. Additionally, skeletal relationships showed a significant relationship with the shape of ST in the study population.

## Introduction

Sella turcica (ST) is a bony structure that is frequently situated in the middle cranial fossa. It is, in fact, a part of the sphenoid bone near the typical anatomical depression. Owing to its general shape, the term ‘sella turcica’ is Latin for ‘Turkish saddle’ [1]. ST is widely utilised as a significant landmark on radiographic platforms such as cephalometrics. In cephalometrics, ST is located on the tracings at the beginning of most analyses. Many researchers have confirmed that ST is situated in the middle cranial fosse. ST is bounded posteriorly by the dorsum sella and anteriorly by the tubercle sella [2]. The lower frontal part of ST is popularly known as the hypophysial fossa. This hypophysial fossa encloses the pituitary endocrine gland. Many researchers agree that ST has complex morphology and anatomical relationships with nearby structures such as the pituitary gland, the internal carotid artery, and the second cranial nerve. Global data on cranial health and trauma confirms that most head injuries, endocrine problems, and tumours are seen in the middle cranial fossa [3].

In the literature, several shapes of ST have been identified by different researchers. However, only six shapes have received worldwide acceptance. They include ST normal, oblique anterior wall, double-contoured sella, a posterior wall with notching, pyramidal shape, and bridged ST [4]. In the developmental stage, ST starts from neural crest cells. A few researchers have claimed that ST develops due to nearby mesoblastic processes [5]. The embryonic development of the pituitary gland runs parallel to ST. However, the pituitary gland matures well before ST [6]. An aberration in the growth progression of the pituitary gland may be the cause of deviance in the anatomy of ST. Any pathology in this vicinity is related to changes in the size of the ST region. Anatomical and morphological variations in ST are frequently associated with the structure’s overall growth and genetics [7-9]. Interestingly, a literature search revealed that there is very little research on the morphology and dimensions of ST. Additionally, clinicians have to rely on conventional measures for the morphometric evaluation of ST in cases of craniofacial abnormalities and syndromes [10-12]. In recent times, cephalometric radiography has been extensively utilised for the diagnosis and prognosis of disorders related to the pituitary gland and ST. Thus, this study is aimed to investigate the discrepancy in the size and shape of ST on the cephalometrics platform based on age and gender in the population of Sri Ganganagar, India.

## Materials and methods

**Figure 1:**
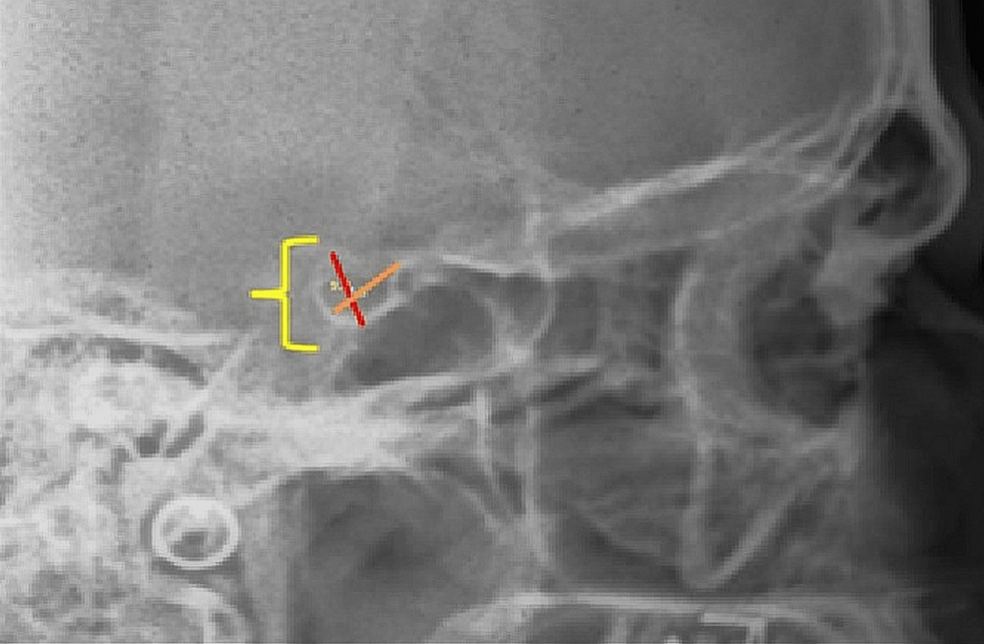
Lateral cephalometric radiograph (with lines marked on the sella; red for depth and orange for length).

This study was conducted in the Department of Oral Medicine and Radiology, Surendra Dental College and Research Institute, Sri Ganganagar. Related searches were conducted from 1 January 2020 to 31 March 2020. A simple systematic sampling procedure was performed to ensure the representativeness of the study sample. Thus, a total of 180 participants of both genders were selected from the patients who visited the outpatient clinic of this department. All participants were aged between 15 and 30 years. The samples were factually selected from the available target population and the accessible population based on clinical, demographic, geographical, and time factors. The 180 participants were equally divided into three age groups: Group I had 60 participants, including 30 male and 30 female participants, aged 15-20 years; Group II had 60 subjects, including 30 male and 30 female participants, aged 20-25 years; and Group III had 60 subjects, including 30 male and 30 female participants, aged 25-30 years. Informed and signed consent was obtained from all participants. This study was approved by the institutional ethical committee’s approval (IEC/21/cmltv215). The following inclusion criterion was followed in the sampling stage: healthy patients aged 15-30 years without any history of systemic diseases and lateral cephalograms with adequate perceptibility of all radiographic landmarks. Patients referred from the orthodontic department for lateral cephalogram radiographs were clinically evaluated, and their demographic information and general and medical history, as well as clinical examination, were recorded (Figure 1).

All patients were subjected to a lateral cephalogram in the natural head position by a trained maxillofacial radiologist using a Kodak 8000CS OPG/lateral cephalometric machine. A single operator manually obtained the cephalometric tracings on standard tracing paper to avoid any possible variation. As per the guidelines of the Steiner analysis, the facial-skeletal relationship is confirmed based on the measured ANB angle. At the beginning of the tracing, the ANB’s typical cephalometric landmarks were identified and marked. Based on these ANB angles, all radiographs were categorised into various classes (class I, II, and III). ANB angles of 20, >40, and 00 (range 00-40) were categorised as class I, II, and III, respectively. Wits analysis was also performed on all radiographs to eliminate any possibility of recording errors. The boundaries of ST were identified and marked cautiously on each lateral cephalogram. The tubercle, floor, dorsum part, and frontal and back clinoid processes dominated the ST outline. Five common variants of ST were noted apart from its typical morphology. The span of ST was noted and recorded on each radiograph using a predefined scoring scale. The length of ST was calculated by marking the points on the tubercle portion and the apex of the dorsum portion. The relative depth of ST was estimated by marking a tangent through the innermost part of ST’s floor. The diameter in the anteroposterior (AP) direction was estimated by measuring the distance between the anterior boundary (tubercle) and the posteriormost point on the distal surface. To avoid any inter-observer discrepancy, all estimations were performed single-handedly at a preset magnification of 1:1 for all samples. The data thus obtained was tabulated and subjected to statistical analysis using the software SPSS (Statistical Package for the Social Sciences) version 21.0 (Microsoft Corporation Inc., Chicago, USA).

## Results

In the study sample (n = 180), 50% were men and 50% were women. The study population was divided into three equal groups (n = 60; 33.33%, each) based on age. The age group ranges (Group I, Group II, and Group III) include 15-20 years, 21-25, and 26-30 years, respectively. The mean length, depth, and AP diameter of the ST of participants in Group I were found to be 9.65 mm, 8.63 mm, and 11.35 mm, respectively. Similarly, for participants in Group II, the mean length, depth, and AP diameter of ST were 9.63 mm, 8.74 mm, and 11.68 mm, respectively. In Group III, the mean length, depth, and AP diameter of the ST were 9.89 mm, 9.27 mm, and 13.05 mm, respectively. The mean length of ST in the lateral cephalogram ranged between 2.91 mm and 3.52 mm for males and between 3.02 mm and 3.53 mm for females.

The mean depth of ST in the lateral cephalogram ranged between 2.59 mm and 3.21 mm for male participants and between 2.67 mm and 3.21 mm for female participants. The mean AP diameter of ST in X-ray ranged between 3.42 mm and 4.48 mm for male participants and between 3.66 mm and 4.21 mm for female participants. The most common skeletal type in the study was class I with 61.70% occurrence, followed by classes II and III with 30% and 8.30% occurrences, respectively. Class I was the most common in the age group 21-25 (60%), while class 2 was the least common (3.33%). The percentage of shapes of ST in both gender groups were as follows: normal 43.3%, oblique 28%, bridging 8.3%, double contour 5%, notching 8.3%, and pyramidal 6.67%. This study indicated that most of the participants had normal-shaped ST. The oblique shape was the second most common shape found in the study population. Male participants showed a predominantly normal-shaped ST, while female participants showed an oblique-shaped ST (Table 1).

 

**Table 1:** Allocation of morphometric shape variation of sella turcica in different age ranges.

Sella turcica shape	Age group (%)			Total
	15-20	21-25	26-30	
Normal	27 (45)	27 (45)	24 (40)	78
Oblique	18 (30)	15 (25)	18 (30)	51
Bridging	3 (5)	6 (10)	6 (10)	15
Double contour	3 (5)	3 (5)	3 (5)	9
Notching	6 (10)	6 (10)	3 (5)	15
Pyramidal	3 (5)	3 (5)	6 (10)	12
Total	60 (100)	60 (100)	60 (100)	180

The descriptive analysis results of the morphometric dimensions of ST is tabulated in Table 2.

**Table 2:** Descriptive analysis results of morphometric dimensions of sella turcica. ST: Sella turcica

Variables		N	Mean	Standard deviation	Standard error	95% confidence interval for mean		Minimum	Maximum
						Lower bound	Upper bound		
ST length	15-20	60	9.6525	2.21199	.28557	9.0811	10.2239	5.62	14.91
	21-25	60	9.6340	1.90811	.24634	9.1411	10.1269	6.76	12.07
	26-30	60	9.8995	2.43122	.31387	9.2714	10.5276	5.26	15.32
	Total	180	9.7287	2.18536	.16289	9.4072	10.0501	5.26	15.32
ST depth	15-20	60	8.7490	1.97723	.25526	8.2382	9.2598	5.34	11.98
	21-25	60	8.6350	1.57451	.20327	8.2283	9.0417	6.12	11.43
	26-30	60	9.2770	1.20220	.15520	8.9664	9.5876	6.57	11.18
	Total	180	8.8870	1.63118	.12158	8.6471	9.1269	5.34	11.98
ST diameter	15-20	60	11.3595	2.73341	.35288	10.6534	12.0656	5.05	15.02
	21-25	60	11.6855	2.39239	.30886	11.0675	12.3035	8.23	16.01
	26-30	60	13.0595	1.93077	.24926	12.5607	13.5583	9.81	17.34
	Total	180	12.0348	2.47460	.18445	11.6709	12.3988	5.05	17.34

An analysis of variance (ANOVA) test revealed a significant difference in the means of the AP diameter of ST among the three age groups. As age increased, the AP diameter increased (Table 3).

**Table 3:** ANOVA test to compare the means of the morphometric dimensions of sella turcica among the study's three age groups. ST: Sella turcica

		Sum of squares	df	Mean square	F	Sig.
ST length	Between groups	2.637	2	1.318	.274	.761
	Within groups	852.231	177	4.815	0	0
	Total	854.868	179	0	0	0
ST depth	Between groups	14.079	2	7.039	2.696	.070
	Within groups	462.197	177	2.611	0	0
	Total	476.275	179	0	0	0
ST diameter	Between groups	97.683	2	0	8.658	.000
	Within groups	998.449	177	5.641	0	0
	Total	1096.132	179	0	0	0

The ANOVA test, however, showed no significant difference in the means of any dimension of ST between the two gender groups. No significant size difference was present in the male and female participants, as shown in Table 4.

**Table 4:** ANOVA test to compare the means of morphometric dimensions of sella turcica between the two gender groups. ST: Sella turcica

		Sum of squares	df	Mean square	F	Sig.
ST length	Between groups	1.502	1	1.502	.313	.576
	Within groups	853.367	178	4.794	0	0
	Total	854.868	179	0	0	0
ST depth	Between groups	.001	1	0	.000	.983
	Within groups	476.274	178	2.676	0	0
	Total	476.275	179	0	0	0
ST diameter	Between groups	.002	1	0	.000	.985
	Within groups	1096.130	178	6.158	0	0
	Total	1096.132	179	0	0	0

## Discussion

ST is an intracranial bony structure that plays an important role in the endocrinal equilibrium of the body. This is because of the presence of the pituitary endocrinal gland inside it. Any pertinent and notable variations in the dimension of ST usually lead to the malfunctioning of the pituitary gland and its hormones. The typical size of ST differs according to the individual’s ethnicity, geography, and race [13,14]. Many studies have shown that the altered morphology of ST is usually associated with some severe pathological conditions, including pituitary tumours, hypoactive pituitary, Down syndrome, and Williams syndrome [15-17]. Srinivas et al. [19] also agreed on the correlation between the altered morphology of ST and the presence of related pathological conditions. These researchers conducted a radiographic study in the Saudi population and found the average length, depth, and AP diameter of ST were 10.85 mm, 9.1 mm, and 13.95 mm, respectively [18,19]. A few other pioneer works have reported somewhat similar measurements (e.g. the length, depth, and AP diameter of ST were 11.35 mm, 9.9 mm, and 13.90 mm, respectively) [6,7,20]. In this study, the results indicated that with an increase in age, there is an increase in ST dimensions. These findings are highly comparable to the study of Sathyanarayana et al. [21], who showed that the ST length tends to increase with age progression but not statistically significantly. Through a Pearson correlation test, Kjær et al. [22] found that the relationship between age and linear dimensions (depth and diameter) is statistically significant. The estimated p-value was highly significant for this relationship (P = 0.001). In this study, the length, depth, and AP diameter gradually increased from 9.63 mm to 9.89 mm, 8.63 mm to 9.27 mm, and 11.35 mm to 13.05 mm, respectively. These findings are comparable to the study by Muhammed et al. [23] who found that the relative volume of ST tends to increase with an increase in age in both genders. It is critical to note that the increase in the ST size could be due to increasing hypophysis [24,25].

Usman et al. [26] found that the normal shape was the most common form of ST in their studied population. The findings in this study are consistent with other studies where the normal shape was also the most common form of ST, with the oblique shape being the second most common variation morphology of ST [27,28,29,30]. In Usman et al. [26], the mean length, depth, and AP diameter of ST in male participants were 9.82 mm, 8.89 mm, and 12.93 mm, respectively. The values for female participants were slightly lesser at 9.64 mm, 8.28 mm, and 12.03 mm for length, depth, and AP diameter, respectively. These findings are inconsistent with Kumar et al. [31], where the measurements of ST dimensions for female participants were larger. However, they are comparable to the findings of Subhadra and Baburao [29] and Campero et al. [30], where the measurements of linear dimensions of ST were slightly smaller for female participants compared to their male counterparts. Various other prominent studies have also shown similar results [30,32,33,34]. Our study confirmed a normal sella shape in nearly 53% of the female participants and an oblique shape in 36% of the male participants. Further, the normal shape is most common in classes I and II. These findings are consistent with Chilton et al. [34], in which class I skeletal relations are predominant and have a normal ST shape, which is the most common anatomy of ST. Silverman [35] also supported this theory and confirmed that the size of Sella enlarges gradually with age. In this study, it was shown that the most common shape of ST was the normal shape in all age groups at nearly 43%, followed by the oblique shape (28%). Hence, sella gives important indications about the pathology in the pituitary gland and helps in the early diagnosis of the different types of lesions (e.g. various adenomas, carcinomas) associated with adenohypophysis or neurohypophysis.

Addressing the limitations, the authors stated that they only utilised two-dimensional radiographic measures instead of advanced technologies such as cone-beam computed tomography. They also studied the intended objectives in localised patients, and therefore, the results of the study may not be applicable to a generalised population.

## Conclusions

This study provided highly imperative presumptions. It confirmed that sella’s anteroposterior dimension is significantly associated with age progression and that the linear morphology of ST has no gender significance. Additionally, the second most common shape of ST after normal morphology was an oblique shape in the study population with no apparent gender differences. Considering the limitations, the authors suggest that other long-term studies be performed using advanced technologies and larger sample sizes to establish authentic guidelines in forensic odontology and other fields of dentistry.
